# Zn–0.8Mg–0.2Sr (wt.%) Absorbable Screws—An In-Vivo Biocompatibility and Degradation Pilot Study on a Rabbit Model

**DOI:** 10.3390/ma14123271

**Published:** 2021-06-13

**Authors:** Karel Klíma, Dan Ulmann, Martin Bartoš, Michal Španko, Jaroslava Dušková, Radka Vrbová, Jan Pinc, Jiří Kubásek, Tereza Ulmannová, René Foltán, Eitan Brizman, Milan Drahoš, Michal Beňo, Jaroslav Čapek

**Affiliations:** 1 Department of Stomatology—Maxillofacial Surgery, General Teaching Hospital and First Faculty of Medicine, Charles University, 121 08 Prague, Czech Republic; karel.klima@vfn.cz (K.K.); dan.ulmann@vfn.cz (D.U.); martin.bartos@vfn.cz (M.B.); michal.spanko@vfn.cz (M.Š.); radka.vrbova@vfn.cz (R.V.); tereza.ulmannova@vfn.cz (T.U.); Rene.Foltan@vfn.cz (R.F.); eitan.brizman@vfn.cz (E.B.); drahomil@gmail.com (M.D.); michal.beno@vfn.cz (M.B.); 2 Department of Anatomy, First Faculty of Medicine, Charles University, 121 08 Prague, Czech Republic; 3 Department of Pathology, First Faculty of Medicine, Charles University, 121 08 Prague, Czech Republic; jaroslava.duskova@lf1.cuni.cz; 4 Department of Functional Materials, FZU The Institute of Physics of the Czech Academy of Sciences, Na Slovance 1999/2, 182 21 Prague, Czech Republic; pinc@fzu.cz; 5 Department of Metals and Corrosion Engineering, University of Chemistry and Technology, Technická 6, 166 28 Prague, Czech Republic; Jiri.Kubasek@vscht.cz

**Keywords:** bioabsorbable metals, in-vivo biocompatibility, magnesium, zinc, strontium, toxicity, biocompatibility, systemic reactions, alloy accumulation, internal organs

## Abstract

In this pilot study, we investigated the biocompatibility and degradation rate of an extruded Zn–0.8Mg–0.2Sr (wt.%) alloy on a rabbit model. An alloy screw was implanted into one of the tibiae of New Zealand White rabbits. After 120 days, the animals were euthanized. Evaluation included clinical assessment, microCT, histological examination of implants, analyses of the adjacent bone, and assessment of zinc, magnesium, and strontium in vital organs (liver, kidneys, brain). The bone sections with the implanted screw were examined via scanning electron microscopy and energy dispersive spectroscopy (SEM-EDS). This method showed that the implant was covered by a thin layer of phosphate-based solid corrosion products with a thickness ranging between 4 and 5 µm. Only negligible changes of the implant volume and area were observed. The degradation was not connected with gas evolution. The screws were fibrointegrated, partially osseointegrated histologically. We observed no inflammatory reaction or bone resorption. Periosteal apposition and formation of new bone with a regular structure were frequently observed near the implant surface. The histological evaluation of the liver, kidneys, and brain showed no toxic changes. The levels of Zn, Mg, and Sr after 120 days in the liver, kidneys, and brain did not exceed the reference values for these elements. The alloy was safe, biocompatible, and well-tolerated.

## 1. Introduction

A vast improvement in living standards over the past few decades, has brought with it increasing demands to medicine. The classical concept for treatment of fractures utilizing steel or titanium plates and screws to stabilize a fractured bone brought with it the need for a secondary procedure to remove these metallic materials from the body. To avoid this, experimentation began with the use of resorbable polymer materials. They are based on poly-lactic or poly-glycolic acids and their co-polymers. Units of polymers are broken down into water and carbon dioxide in the body. Some patients exhibit an inflammatory reaction during the process of resorption. In normal conditions, bending these polymer plates is not possible. Only hot water or ultrasound can be used for the bending and thus better alignment of polymeric materials. The load-bearing ability of polymers is also low, which represents another disadvantage. Absorbable metals could solve these problems with: (i) better bending for individualization of bone shape, (ii) better mechanical strength, (iii) possibly better degradation parameters after fulfilling their function by different principles of absorption [1,2,3].

Biodegradable metals (BMs) are defined by gradual corrosion in-vivo with appropriate reaction of a host body. BMs should dissolve completely when fulfilling their mission. Therefore, a major component of BMs should be elements that could be metabolized by a human body without toxic, carcinogenic, teratogenic, and allergic reactions [4]. The international organization for standards (ASTM) has defined standards for bioabsorption of absorbable metals (F3160 and F3268) [5,6].

The history of resorbable metals began in 1878 when Edward C. Huse used magnesium wires for ligature of bleeding vessels [7,8]. He described that the resorption rate depended upon the diameter of the Mg wire. Austrian physician Erwin Payr continued by using magnesium in various surgical procedures. He developed a magnesium connector for end-to-end vessel anastomosis. Payr also described an intramedullary stabilising rod for the treatment of non-union fractures [7]. Magnesium-based materials with absorbable properties have been extensively studied since the turn of 20th century and have been used for the treatment of (i) vessel stenting [9], (ii) intestinal anastomoses [7], and (iii) bone implants [1,7,10], both in experimental animals [2,3,11,12] and also in humans [1,10]. Even though Mg-based materials are now readily used in surgical practice [10,13,14], they still possess some drawbacks—preventing their wide use in surgery. In particular, their corrosion rate is too high for many applications. Moreover, the corrosion process is accompanied by hydrogen production, which may have a detrimental effect on the healing process [15,16]. Due to that side effect, iron- and zinc-based materials have been suggested as alternative potential metallic absorbable implants for both cardiovascular and bone implants [15,16,17]. In contrast to magnesium, iron and its alloys degrade very slowly. Except for the austenitic alloys, the Fe-based alloys are ferromagnetic, which precludes patients from undergoing magnetic resonance imaging. This limits the application of Fe-based alloys in implantology as well [18,19]. In 2011, Vojtěch et al. introduced zinc as a candidate for fabrication of biodegradable implants [13,14,20]. Since that time, many Zn-based materials containing a large variety of different alloy elements have been investigated. Based on the obtained results, it was suggested that Zn-based materials can adequately fulfil all requirements for use in implantology [16,18,19,21].

In this paper, we show the results of in-vivo testing of Zn–0.8Mg–0.2Sr (wt.%) implants—in the form of maxillofacial screws in rabbit tibias—left exposed to their environment for 120 days. The biocompatibility, degradation, and implant–bone interaction were investigated. To allow the readers an easy comparison with those properties of other Zn- and Mg-based absorbable materials, a short review of the results obtained by various scientific teams was performed. The results of this review are summarized in Table 1 and in the following paragraphs.

*The In-Vivo Biological Behaviour of Zn- and Mg-Based Materials*

From the aforementioned studies, which were analysed in detail and whose results are summarized in Table 1, magnesium can be characterized as having very good biocompatibility with the surrounding bone—expressed by good new-bone production, good osteoconductivity and osteoinductivity, and good mechanical properties [2,3,27,28,30]. In the case of pure magnesium, hydrogen pockets were often formed [2,28,29]. Those gas pockets did not interfere with normal bone healing or lead to any significant inflammation or necrosis of the surrounding soft tissues [3,28,29]. In one study that used pure Mg, contamination around the bone by corrosion products was observed [3], and in a study where the author used an alloy of Mg–4Li–3.6Al–2.4REE (wt.%), REE contamination around the implanted alloy and in vital organs was reported [12]. The use of pure Mg metal did not significantly increase the level of Mg in the blood or surrounding organs [11,26,27,28]. The rate of corrosion and resorption of the experimental metal was faster for pure magnesium [3] compared to magnesium alloys with the addition of Sr [25].

Zinc alloys also show good mechanical properties [22,23,24] with good biocompatibility and no increase in Zn and Sr levels in vital organs compared to the use of pure Ti [22]. The corrosion rate of a zinc alloys is slower compared to magnesium alloys. Pure Mg resorbs at a rate of 25% volume reduction in 12 months [26], whereas pure Zn resorbs at a rate of 10% per year, equivalent to only 0.14 ± 0.05 mm/year [21,23]. Zinc alloys exhibited similarly slow resorption rates: Zn–2.0Mg–2.0Fe alloy had a degradation rate of 0.095 ± 0.009 mm/year [24]. One study observed an insignificant increase in serum Zn levels compared to the post-op and pre-op levels [24]. In recent years, Zn alloys have been preferred for their slower degradation rate, as well as not having any production of hydrogen gas pockets and their good mechanical and biological properties. We decided to use a Zn–Mg–Sr biodegradable alloy for our experimental rabbit study. This alloy was used because of the results that we obtained in our previous studies that described the mechanical, corrosion, and in-vitro biological behaviour of a Zn–0.8Mg–0.2Sr extruded alloy [31,32]. Those studies showed that this alloy possesses promising behaviour for application as a bone implant [31,32].

## 2. Materials and Methods

### 2.1. Implant Material

The extruded Zn–0.8Mg–0.2Sr (wt.%) alloy was prepared by melting a mixture of appropriate amounts of pure elements followed by gravity casting. Subsequently, the ingot was annealed for 24 h at a temperature of 350 °C to homogenize its composition and microstructure. The annealed material was extruded at the temperature 200 °C using an extrusion ratio of 25:1. The preparation procedure is described in more detail in our previous study [32]. The exact chemical composition of the alloy was measured via atomic absorption spectroscopy (AAS) using an Agilent 280FS AA spectrometer (Agilent, Santa Clara, CA, USA) with flame atomization. For this analysis, samples from several locations of the extruded rod were dissolved in HNO_3_ and the obtained solutions were diluted with deionized water to concentrations suitable for analysis. The mean composition of the alloy was as follows: 0.83 wt.% of Mg, 0.17 wt.% of Sr, and 99 wt.% of Zn.

**Figure 1:**
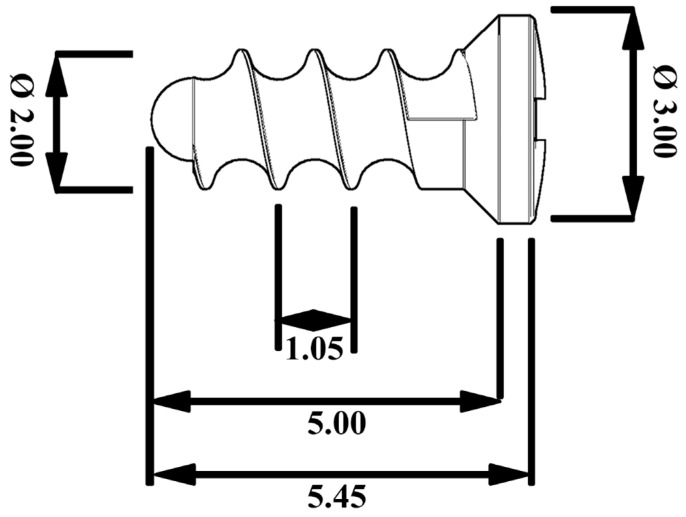
A schematic figure of the screws used in this study. The dimensions are listed in mm.

The extruded rods were machined using a CNC Fanuc Robodrill α-T21iFa machine (Fanuc, Tsukuba, Japan) into screws whose shape and dimensions were inspired by so called “micro-maxillofacial” screws. The shape of the screws was designed according to the mechanical properties of the selected alloy to withstand expected loading during implantation and subsequent exposure in tissue. A scheme of the screw is shown in Figure 1.

### 2.2. Animals

We used 3 male New Zealand rabbits (Velaz, Prague, Czech Republic) with body weights 750–850 g. We only included males in our study to minimize the effects of hormone levels on the variability of the healing as well as bone regeneration [33,34]. This study was performed in accordance with the European Communities Council Directive of 24 November 1986 (86/609/EEC) regarding the use of animals in research and was approved by the Ethical Committee of the First Faculty of Medicine, Charles University, Prague, Czech Republic. All effort was made to minimize the number of animals used in the study.

### 2.3. Experimental Group

Three laboratory rabbits were chosen at random to have the experimental alloy screw implanted into their tibia bone.

### 2.4. Surgery

The implant procedure was performed on the rabbits under general anaesthesia. All surgery was performed in a certified veterinary operating theatre. Anaesthesia was induced with 5% isoflurane (Isoflurane Piramal, Piramal Healthcare UK Ltd., Morpeth, UK) at a flow rate of 300 mL/min. The animals were maintained with ketamine 20 mg/kg and xylazine 3 mg/kg. Hair was shaven from the tibia bone region under aseptic conditions and then a 2 cm skin incision at the proximal part of diaphysis of the tibia bone was made. The muscles and periosteum were reflected from the bone. A hole was drilled into the proximal diaphysis of the tibia bone to a depth of 6 mm with a diameter of 1.6 mm. The drilling was accompanied by sterile cooling utilising a physiological solution. The three rabbits were implanted with an experimental alloy screw measuring 2 mm in diameter and 5 mm in length. All layers were sutured by resorbable sutures. The rabbits were euthanized 120 days after the surgery.

### 2.5. Euthanasia

Euthanasia was performed by inhalation of the anaesthetic isoflurane (Isoflurane Piramal, Piramal Healthcare UK Ltd., Morpeth, UK) that is otherwise used for general anaesthesia. Subsequently, 360 mg sodium thiopental (Sandoz GmbH, Vienna, Austria) was injected directly into the heart, leading to immediate cardiac failure. After reflection of the soft tissues, we harvested the bone specimens containing the experimental screws. The bone, together with specimens of the liver, kidneys, and brain, were fixed in 4% formalin solution and processed for histological examination. The bone was also examined via microCT. Liver, kidney, and brain specimens were frozen to −40 °C for analysis of strontium, zinc, and magnesium levels.

### 2.6. Histological Methods for Analysis of the Bone Specimens

The tibiae were fixed in a solution of 36–38% formaldehyde and 80% ethanol (ratio 1:2). The samples were dehydrated for 1 week in a series of ethanol solutions ranging from 70 to 100%, 1 concentration/1–2 days, culminating with 1 day in a solution of 100% ethanol and acetone (ratio 1:1). The bones were twice immersed in destabilized methyl methacrylate (MMA; 1 MMA/2–3 days), and finally in an embedding medium (100 g MMA + 12 mL dibutyl phthalate + 1.8 g benzoyl peroxide). Penetration of the medium was aided by a vacuum pump (ILMVAC GmbH, Ilmenau, Germany). Polymerization was induced using a water bath (thermostat F25-HE, Julabo, Seelbach, Germany) with the temperature rising by one degree every 2–3 days from 24 to 36 °C. The bone blocks were cut using a lab saw ISOMETTM with a diamond disc (Buehler, Lake Bluff, IL, USA). The surfaces were ground down using wet silicon–carbide papers P1200, P2500, and P4000 and polished with 1 mm and 0.3 mm Al_2_O_3_ suspensions used in combination with TexMet and MicroCloth polishing cloths (Buehler, Lake Bluff, IL, USA) and the equipment MetaServ 250 (Buehler, Lake Bluff, IL, USA). The sections of 60 µm thickness were stained for 5 min with 1% solution of toluidine blue in 30% ethanol and heated to 60 °C. After rinsing with running distilled water and differentiation in 96% ethanol, the slides were stained for 12 min with 0.2% solution of toluidine blue in a phosphate buffer (pH = 9.1) and heated to 60 °C. The slides were then washed with distilled water and dried. The healing process of the tested materials in the artificially created holes and grooves was examined via optical microscopy using a Nikon Eclipse 80i microscope (Nikon Instruments Inc, Melville, NY, USA), Jenoptik camera (Jenoptik, Jena, Germany), and an image analysis system by NIS Elements, Nikon AR (Nikon Instruments Inc, Melville, NY, USA). Any bone reaction, its morphology, and the presence of fibrous tissue and cells were studied and evaluated on the histopathology of specimens using a semiquantitative scoring system with parameters described by Reifenrath et al., 2011 [35]. The evaluated features were: gas bubble formation, overall bone structure, bone cavities, periosteum remodelling, endosteal remodelling, periosteum apposition, peri-implant bone formation, peri-implant fibrosis, lymphoplasmocellular reaction, presence of macrophages, and giant cells [35]. Two to five histological slides from each implant were examined. Any morphological signs of possible damage and bone tissue response were monitored at the level of: (i) periosteum, (ii) endosteum, (iii) bone–implant contact (BIC) [36,37], (iv) connective tissue formation, (v) inflammatory response, and (vi) ossification in the newly formed connective tissue [35].

### 2.7. Histopathological Methods of Parenchymal Organ Processing

The organs of the sacrificed rabbits were fixed in a buffered formalin solution. Macroscopically, they did not exhibit any pathology changes. Two excisions from the kidney and liver and a part of the brain tissue were embedded in paraffin. Standard haematoxylin & eosin staining was used, and in some samples Pearls’ reaction for iron was also performed. In all animals, kidney, liver, and brain specimens were investigated for any possible toxic influence of the implanted material.

### 2.8. Methods of X-ray Examination

All rabbits underwent X-ray examination under general anaesthesia 120 days after implantation. All surgery was performed in a certified veterinary operating theatre. After induction of anaesthesia with 5% isoflurane (flow 300 mL/min), we made two projections of each rabbit’s tibia, one projection perpendicular to the other. All X-rays were made using an In-Vivo Xtreme BI 4MP (Bruker BioSpin, Rheinstetten, Germany). In order not to disturb the bone around the experimental screw, the X-ray examination was performed immediately prior to sacrificing the animals.

### 2.9. MicroCT Examination

The bone specimens (n = 3) containing the implants were scanned using a desktop SkyScan 1272 Micro-CT (Bruker, Kontich, Belgium). The specimens were immersed in 70% ethanol solution and scanned in plastic tubes with the following parameters: pixel size 15 μm, source voltage 100 kV, source current 100 μA, 0.11 mm Cu filter, frame averaging 3, rotation step 0.1°, rotation 180°. The scanning time was approximately 2 h for each specimen. The flat-field correction was updated before each acquisition. Image data were reconstructed and processed using NRecon, DataViewer, CTVox, and CTAn software (Bruker BioSpin, Rheinstetten, Germany). Prior to 3D analysis of the implants, the parametric data for the volume and surface values were image-processed (with respect to improvement of the signal-to-noise ratio) and subsequently binarized. Data processing was optimized using TeIGen software, which is described in [38]. Bone–implant contact was quantified via manual measurements of the 2D cross-section images in each specimen (BIC = implant perimeter in contact with bone-implant perimeter). The bone contact was only evaluated in the cervical region of the screw because the implant apex position was quite variable amongst the specimens and the screw head was excluded from this evaluation.

### 2.10. Analysis of the Solid Corrosion Products

To investigate the extent and uniformity of the corrosive process and the chemical composition of the solid corrosion products, sections of the bones with implanted screws were observed using a scanning electron microscope FEI Quanta 3D FEG (ThermoFisher Scientific, Waltham, MA, USA) equipped with an energy dispersive spectrometer EDAX Apollo 40 (Ametek, Berwyn, PA, USA) (SEM-EDS).

### 2.11. Analysis of Systemic Toxicity in the Vital Organs

Liver, kidney, and brain specimens were analysed for their content of strontium, magnesium, and zinc.

The tissue samples from the experimental animals were mineralized utilizing nitric acid and hydrogen peroxide. The mineralization process would cause decomposition of the biological and inorganic matrix, enabling the transfer of the analytes into a solution which would allow for their analysis. A Milestone MLS 1200 MEGA microwave (Milestone Inc., Shelton, CT, USA) mineralization device with a 6-position high-pressure decomposition rotor and an evaporating rotor was used to mineralize the samples. Control reference materials and blank samples were mineralized together (in parallel) with the actual samples.

### 2.12. Strontium and Zinc Analysis

The content of Sr and Zn elements in the samples was determined via inductively coupled plasma mass spectrometry (ICP-MS) on an ELAN DRC-e instrument (Perkin Elmer SCIEX, Waltham, MA, USA) in which the concentrations of the given analytes in the sample solution were selectively determined. Strontium ions ^88^Sr and zinc ions ^66^Zn were used for quantification. The sample mineral was adjusted, if necessary, by dilution and addition of a ^74^Ge internal standard for both monitored elements before final analysis via the ICP-MS method. Quantitative evaluation of the analytes was performed with external calibration. Calibration for Sr was in the range 0–10 µg/L and for Zn the range 0–500 µg/L. A Seronorm WB L-2 whole blood sample was used as a control. The limit of detection for Sr was 0.003 µg/g tissue and for Zn 0.1 µg/g tissue.

### 2.13. Magnesium Analysis

The magnesium content was determined in the sample via flame atomic absorption spectrometry (F-AAS) on an AAnalyst 400 (Perkin Elmer, Waltham, MA, USA). Before the F-AAS analysis, the sample was treated by diluting and adding ionic buffers Cs and La so that their final concentration in the solution was 2000 mg/L. Quantitative evaluation of the analytes was performed with a method of external calibration. Calibration for Mg was in the range 0–2 mg/L. A Seronorm WB L-2 whole blood sample was used as a control. The limit of detection for Mg was 0.2 µg/g tissue.

## 3. Results

### 3.1. Mechanical Properties of the Alloy

The mechanical behaviour of the investigated alloy has been studied and was discussed in our previous work [32]. It is worth mentioning that the tensile yield strength, ultimate tensile strength, ductility, and Young’s modulus of the alloy were 244, 324 MPa, 20%, and 104 GPa, respectively.

### 3.2. X-ray Examination

**Figure 2:**
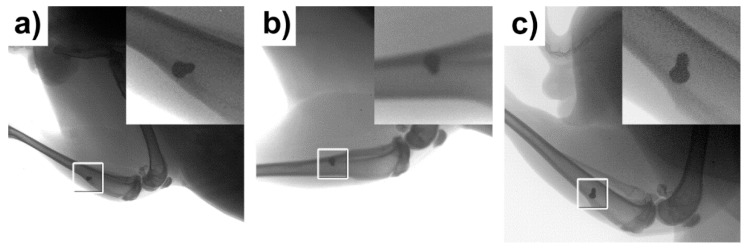
X-ray of the tibial bone of the three experimental rabbits 120 days after experimental screw implantation. (**a**) rabbit no. 1, (**b**) rabbit no. 2 and (**c**) rabbit no. 3. There are no osteolytic changes seen between the bones and experimental screws.

The specimens were visualised using a standardised 2D X-ray imaging technique in two planes 120 days after implantation. The images obtained via X-ray examination are shown in Figure 2. The screws appeared to be well integrated, showing successful osseointegration. Microscopic interposition of fibrous tissue (between the experimental screws and the adjacent bone) could of course not be excluded. No osteolytic changes were seen around the experimental screws indicating that no inflammatory response took place.

### 3.3. MicroCT Examination

**Figure 3:**
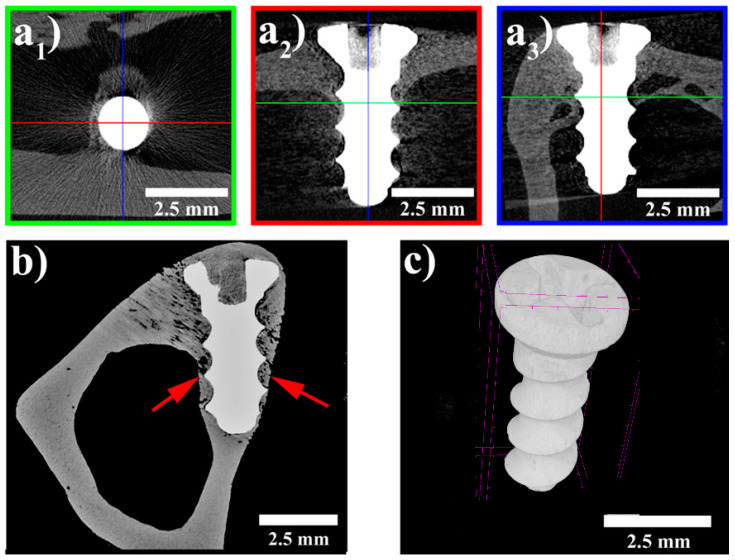
MicroCT images; (**a1**–**a3**) standardised cross-section images in three perpendicular planes; (**b**) implant completely surrounded by bone—arrows; (**c**) 3D visualization of the implanted screw.

The specimens were visualized using a standardized 2D cross-sectional image in 3 perpendicular planes (Figure 3a). The microCT image data enabled quantitative evaluation of bone–implant contact (manual 2D measurement), the quantitative 3D analysis of implant structure, and qualitative evaluation of bone surrounding the implants. However, metal-induced artefacts in the experimental group did not allow quantitative 3D analysis of bone–implant contact (Figure 3a,c).

All the evaluated implants appeared to be in contact with the surrounding bone without any signs of a foreign body reaction or fibrointegration. The thickness of the bone surrounding the implants was found to be greater than the average cortical thickness. Histomorphometric analysis of bone–implant contact was established as the most accurate method for evaluation of the percentage of the implant in contact with bone [36,37]. The mean BIC value for the implants at 120 days was 22%. There were no signs of implant degradation. Only minor changes of volume and surface were found in the screws.

### 3.4. SEM-EDS Observations of the Implant–Bone Interface

**Figure 4:**
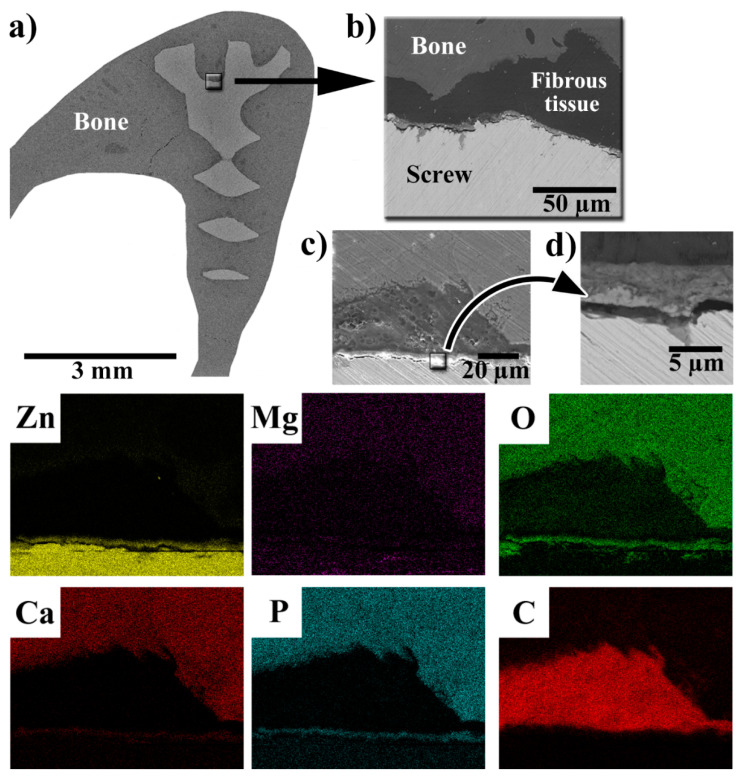
SEM-EDS observation of the bone section with the implanted screw. (**a**) SEM picture of the overview; (**b**) SEM picture of the screw–bone interface; (**c**) SEM picture of the area mapped via EDS (the elemental maps are shown below); and (**d**) a detailed view (SEM) of the layer of corrosion products.

The results of SEM-EDS observations of a section of a bone with an implanted screw are shown in Figure 4. In Figure 4, it is visible that new bone has overgrown the head of the screw (Figure 4a), which suggests good biocompatibility of the implanted material. At higher magnifications, it was observed that the implant was surrounded by tissue rich in carbon. As will be shown later, this tissue was identified as fibrous tissue. In Figure 4d, it is clearly visible that the implanted screw was covered by a layer of solid corrosion products, which consisted of Zn, O, Ca, and P. This suggests that the corrosion products were mainly based on phosphates. The thickness of the layer of corrosion products ranged between 4–5 µm, corresponding to an average degradation rate of approximately 13.5 µm/year.

### 3.5. Histopathological Examination of Bone Specimens Containing Implants

**Figure 5:**
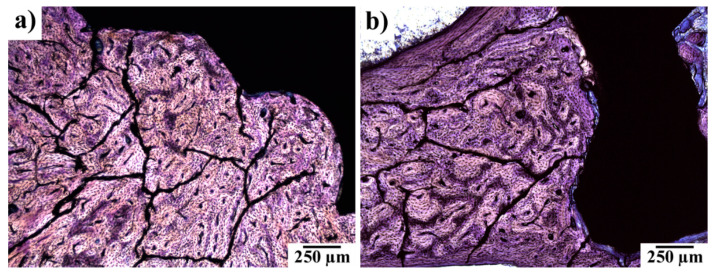
Histological examination of implant site with experimental screw. (**a**) Irregular osteon arrangement; (**b**) regular osteon arrangement. Note: the black field represents a histological cut of an experimental screw. Toluidine blue stained slides.

The presence of gas bubbles was not detected in any of the examined samples. As is shown in Figure 5, the irregular structure of the bone was observed rather rarely in the vicinity of the implants (Figure 5a) and the majority of the bone surrounding the implant was of a regular structure (Figure 5b).

**Figure 6:**
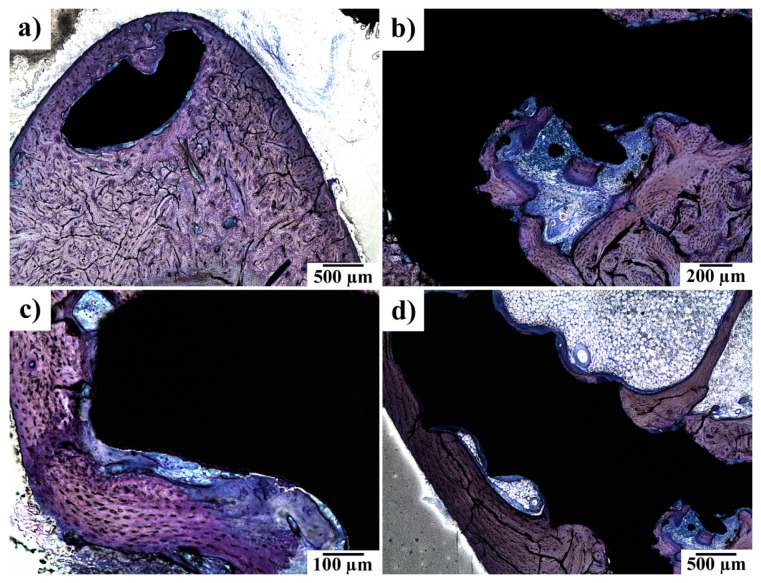
(**a**) Head of the implanted screw (black) embedded in the newly formed bone–periosteal apposition here is followed by regular bone formation; (**b**) periosteal apposition and bone remodelling over an experimental screw; (**c**) bone remodelling with bundle bone formation in the presence of an experimental screw; and (**d**) endosteal remodelling–intrabony segment of the experimental screw. At 120 days after implantation. Toluidine blue staining.

In Figure 6, periosteal apposition and endosteal remodelling in the vicinity of an implanted screw are shown. The periosteal apposition was connected with the remodelling of new bone tissue. Bundle bone was formed more often than the lamellar one. The intensity of endosteal remodelling was less frequent than periosteal apposition, although a similar extent of those events was observed in individual experimental animals.

**Figure 7:**
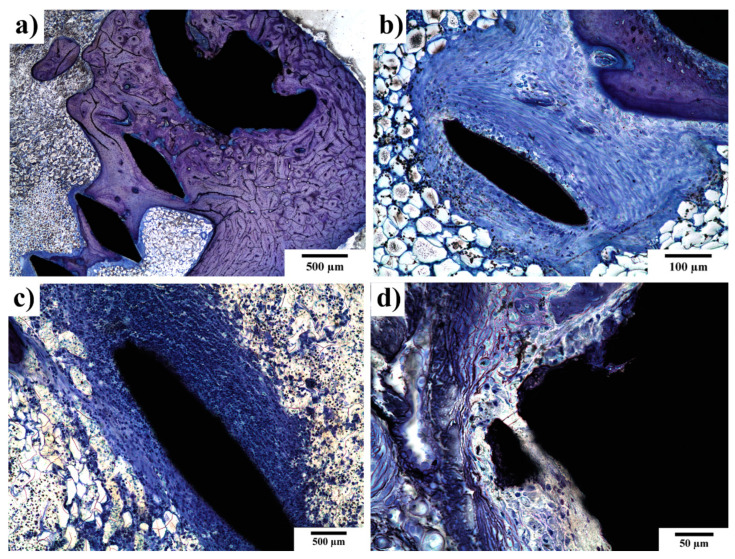
Peri-implant fibrosis around experimental screw 120 days after implantation. (**a**) The whole surface of the experimental screw was covered with fibrosis; (**b**) under high magnification, remnants of fibroproductive inflammation, represented by scattered lymphocytes and macrophages in the fibrous tissue, can be traced; (**c**) peri-implant fibrosis with dense lymphocytic infiltrate surrounding the experimental screw; and (**d**) macrophages in close vicinity to the implanted screw. At 120 days after implantation. Toluidine blue staining.

In Figure 7, one can clearly see that peri-implant fibrosis took place. The fibrous tissue covered more than 51% of the implant surface but with variable thickness. The thickness of the fibrous tissue ranged between 0.01 mm and 0.3 mm (see Figure 7 and Table 2.). At 120 days after implantation, two out of the three rabbits had no detectable inflammatory reaction. The third rabbit reacted, presenting with chronic lymphoplasmocellular infiltration in the peri-implant fibrous tissue. There were scattered macrophages as well; no giant cells were found. As is shown in Figure 7c, the inflammatory response in the connective tissue surrounding the implant was of moderate to high intensity. In contrast, the presence of macrophages was mostly subthreshold (<3 in section) or sparse (3–20 macrophages in section), as is visible in Figure 7d. Neither phagocytosed material in the cytoplasm nor substantial irregularities of the experimental screw surface were observed. Nevertheless, their role in triggering the fibroproductive inflammatory process is undeniable. There were no signs of active osteolysis beyond the described peri-implant fibrosis. Giant multinucleated cells were only found exceptionally—in one solitary case.

**Figure 8:**
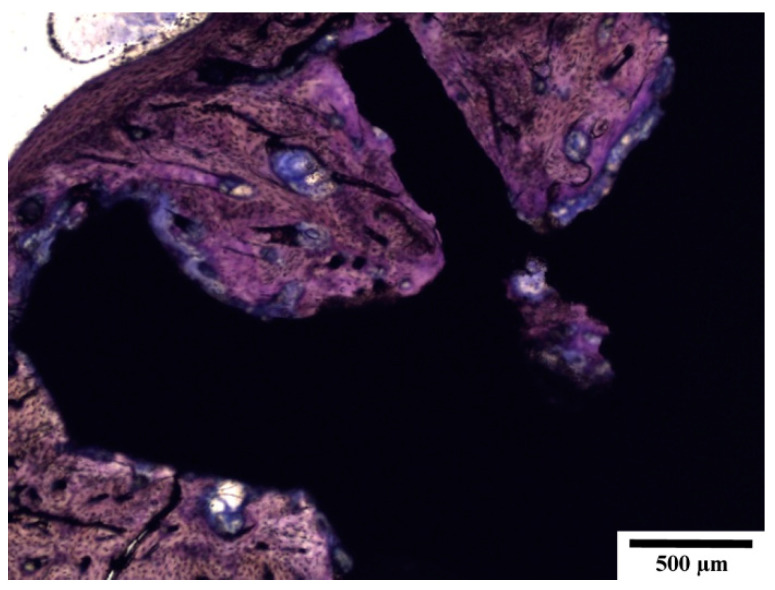
Very minimal disturbance of the contour sharpness showing a low level of resorption.

As is visible in Figure 8, the implanted material showed (at the level of light microscopy) only minimal disturbance to the contour sharpness—as a considered sign of resorption. No metallosis (expressed by a granulomatous reaction) was observed.

**Figure 9:**
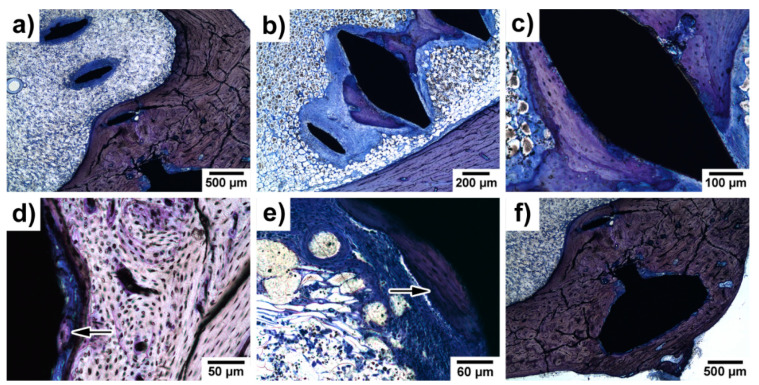
(**a**) Peri-implant fibrosis; (**b**,**c**) bone formation with tight apposition of the newly created bone to the surface of the experimental screw; (**d**) initial bone formation—detailed view; (**e**) fibroproductive inflammation and initial bone formation; and (**f**) thickened compact bone with the experimental screw embedded. Peri-implant bone formation around the experimental screw is marked by arrows in (**d**,**e**). At 120 days of implantation. Toluidine blue staining.

Despite the presence of the fibrous tissue, peri-implant bone formation was always expressed but with markedly variable intensity (Figure 9). The formation of new bone indicates that the experimental alloy has biocompatible properties to bone. Bone regenerates at a slow rate. Thus, 120 days from the insertion of the screw represents an adequate increase of bone around the implants.

A summary of the morphological findings on the tibia of the rabbits is shown in Table 2. The material was well-tolerated. No gas bubbles were observed. Regular structure of the bone surrounding the implanted material in the majority of rabbits was seen, with only focal irregularities observed in 3 out of the 11 slides examined. Bone cavities were not found. Periosteal remodelling and apposition were present with mild or medium intensity. Medium-to-high intensity of endosteal remodelling was also a constant finding in samples with implant/endosteal contact. Peri-implant fibrosis was presented with high intensity (score 3) in all slides with implant/bone interface.

### 3.6. Systemic Toxicity in the Liver, Kidneys and Brain Specimen

**Figure 10:**
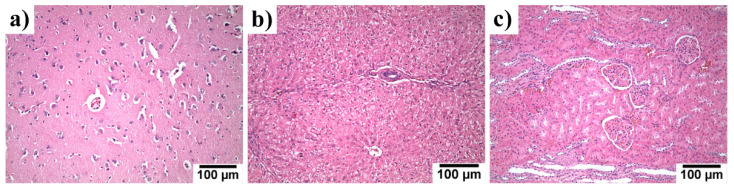
Histological examination of (**a**) brain, (**b**) liver, and (**c**) kidneys of rabbits 120 days after implantation with experimental screws. After 120 days there was no visible morphological/histopathological evidence of any toxic effect in the examined organs from the absorbable metals used. All specimens were processed in the routine way, i.e., paraffin-embedded, 5-µm-thick sections stained with haematoxylin & eosin.

The kidneys, liver, and brain of the experimental rabbits were histologically examined 120 days after experimental alloy implantation. Histological evaluation with haematoxylin & eosin staining of those organs is shown in Figure 10. No morphological signs of toxic damage to the kidneys, liver, and brain were observed.

The content of the elements contained in the implanted material (Zn, Mg, and Sr) in the organs was analysed and the results can be seen in Table. 3. In Table 3, the contents of the elements found in the organs of control groups used in other studies are also listed. The concentrations of Zn and Mg found in the organs of our experimental group was comparable with those of the control groups. For strontium, we found a common Sr content in organs only for rabbit females, whose organ composition could slightly differ from that of rabbit males [39]. In spite of that, the Sr content in livers was comparable to that found in the control group in [25]. The Sr content in the kidneys was higher in our experimental group, but this could be caused by the differences in the gender of the experimental animals [39]. Due to the fact no toxic effects were observed (see Figure 10) and considering the results obtained by Jia et al. [22], who found no increase of Sr in organs of rats after the implantation of a Zn–Sr alloy, we can suppose that only negligible or rather no accumulation of strontium in the organs took place in our case.

## 4. Discussion

Age-related fractures are projected to increase in the U.S. to over 3 million fractures per year by 2025 [44]. In human traumatology, especially paediatric traumatology, the use of absorbable metals represents an interesting alternative to conventional titanium or steel plates and screws. Conventional fracture management often requires removal of the implanted material at a later date. Minimizing the number of operations would be beneficial for the patients and could also bring significant financial savings [1,26]. As shown in our previous study [32], the mechanical and tensile strength of the investigated material fulfilled all the basic criteria for the fabrication of bone implants [45]. Magnesium alloys also have good mechanical properties [3,12], are well tolerated by bone and soft tissues [2,3], but produce gas bubbles [2,3,28], diminishing the possibilities of their practical use in human and veterinary medicine. Therefore, we have chosen a Zn–0.8Mg–0.2Sr alloy, which is known to corrode without hydrogen evolution and fulfil the basic mechanical criteria for implantology [31,32]. Moreover, this alloy was found to possess acceptable cytocompatibility in-vitro and enhanced antibacterial activity [32]. After 120 days of implantation, no volume changes of the screw or formation of voluminous corrosion products were observed via microCT and light microscopy, suggesting a low degradation rate. As was proved by the SEM-EDS analyses, the corrosion was connected by a formation of solid corrosion products based on phosphates. The corrosion products formed a relatively uniform and dense layer, which acts as a barrier and slows the degradation process [31]. Formation of Zn-based phosphates can be beneficial because, as was proved by Su et al., they can show antibacterial behaviour and enhance the biocompatibility of the material [46]. Bonding of the Zn^2+^ ions into solid phosphates also decreases the risks connected with the transport of Zn^2+^ ions to surrounding tissue and to important organs. As a consequence, no osteolysis and no enhanced concentrations of Zn, Mg, or Sr in the examined organs was observed (see Table 3). The degradation rate was comparable with those observed in other in-vivo studies dealing with Zn-based biodegradable alloys for bone applications [21,23,24]. The degradation rate was rather slow considering the basic requirements for bioabsorbable metals. The absorbable implant should degrade 1–2 years after the implantation [47], which would not be fulfilled in our case—considering a constant degradation rate of about 13.5 µm/year. Histologically, no phagocytosed material was observed in the cytoplasm, or were there any substantial irregularities found in the surface of the experimental screws.

Böstman et al., in their human study, observed that absorbable materials from polyglycolide rods would often lead to sterile inflammatory reactions, visible via X-ray as osteolysis—the destruction of bone around an implant [48]. Osteolysis is generally recognised as an indirect indicator of irritant or toxic changes to bone from a foreign material [49]. In our experiment on extruded Zn–0.8Mg–0.2Sr alloy implanted in rabbits, there were no osteolytic changes observed via X-ray. Via microCT, all implants were seen to be in contact with the surrounding bone, with no signs of any adverse reactions. The thickness of the bone surrounding the implants was found to be greater than average cortical thickness, proving biocompatibility. Histologically, we used a proven scoring method [35] to evaluate the regeneration of hard and soft tissues around the experimental screw. Histologically, peri-implant bone formation was observed after 120 days together with peri-implant fibrosis. Zinc supplementation stimulates the osteoblast bone formation and inhibits osteoclast differentiation and results in increased bone strength [50,51]. A large amount of newly formed bone tissue in the zinc alloy group was described in the systematic review and meta-analysis based on animal studies. In addition, the newly formed trabecular bone was also thicker than that in the pure titanium group [52]. Based on that, we can deduce that local zinc enrichment may promote osteogenesis. This could explain the thickened compact bone in the vicinity of the experimental screws (Figure 9f).

Osseointegration means that bone cells (osteoblasts) grow directly onto the surface of an osseointegrated implant [53]. Titanium, for example, has this ability [36,53]. Bone–implant contact was observed in the experimental screws, with peri-implant fibrosis histologically described in more than 51% of surfaces in all experimental specimens. Fibrosis is the reaction of the body to a foreign object [54]. It can be described as a “foreign body reaction “. This is the end-stage response to inflammation, composed of macrophages and foreign body giant cells reacting to wound healing following implantation of a medical device, prosthesis, or biomaterial [44]. Engraftment of an implant in soft tissue is characterized by fibrointegration. The presence of fibrous tissue surrounding Zn-based implants is often observed [22,23,55]. We can conclude that biodegradable alloys could osseointegrate which means that they have a good biocompatibility. We observed good osteogenesis around the implanted screws. Partial fibrointegration was observed in our study, too. The presence of fibrous tissue around an implant was found in fast-degraded ones [56]. By other authors, the bone–implant contact ratio varied depending on the degradation behaviour of implants [23]. Slow corrosion rate resulted in improved BIC, while severe localized corrosion provoked a thick fibrotic layer surrounding the implants [23]. We can conclude that absorbability requires reaction from surrounding tissues. This reaction should not be aggressive and therefore not result in osteolytic changes of the bone or a massive significant inflammatory response. It should be a mild one, which was observed in our study.

We did not detect any white blood cells, indicative of an inflammatory reaction, in the majority of specimens. Only one histological slide (from 15 slides) showed a possible chronic inflammatory reaction. We can therefore conclude that our pilot study showed histologically, after 120 days, that there was very good tolerance to the implanted material. This finding, however, may have been slightly underestimated as we did not use a special staining (TRAP—tartrate-resistant acid phosphatase staining) [57]. TRAP staining would possibly have contributed to a better analysis of the inflammatory response to an implanted experimental alloy.

Similarly, as in other studies investigating Zn-based biodegradable alloys, we observed no damage to the examined organs of the experimental animals. This can be attributed to the low degradation rate and to the fact that the material transformed into solid corrosion products. As a result, only a minimal amount of free metallic ions, which could be transported to the organs and cause their damage, was formed. Such a small amount of free metallic ions was most likely metabolized without any adverse effect on the host organism or accumulation of metallic ions in organs, as was shown in Table 3). The recommended dietary allowances (RDAs) of zinc are 11 mg/day for males and 8 mg/day for females [58]. Tolerable upper intake levels (ULs) for zinc are 40 mg/day [58]. For experimental rabbits, a diet containing at least 50 mg/kg is usually fed [42]. Even if the material was fully soluble and did not form any solid corrosion products, the ion release at the measured corrosion rate (~3.5 µm/year) would be approximately 5 mg/year (considering the surface area of the screw being 52.5 mm^2^, which was estimated from the CAD model of the screw). Such an amount is approximately thousand times lower than the RDA for human males and also lower than the Zn content in the recommended rabbit diet. The recommended dietary allowances (RDAs) for magnesium are 420 mg/day for males and 320 mg/day for females [59]. For rabbits, the usual diet contains about 2.5 g/kg of magnesium. In human adults, the total daily intake of strontium is estimated to be about 4 mg/day [60]. Drinking water contributes about 0.7–2 mg/day and food (mainly leafy vegetables, grains, and dairy products) another 1.2–2.3 mg/day [60]. A maximum acceptable concentration (MAC) of 7.0 mg/L is proposed for total strontium in drinking water [39]. The content of magnesium and strontium in the organs related to other studies [25,40] and was within normal levels. All three used elements are known for promoting bone regeneration [3,22,61,62,63]; therefore, their slow release during the implant degradation could be beneficial for the healing of the injured bone. Based on the obtained data, we can conclude that the implanted materials should not cause any systemic toxicity and organ damage in humans because the amount of released ions were negligible compared to the common daily intake of Zn, Mg, and Sr.

## 5. Conclusions

In this study, Zn–0.8Mg–0.2Sr alloy was successfully implanted into rabbits and monitored for 16 weeks (120 days), which presented a longer interval than in other published studies related to the application of zinc-based materials as bone implants. The screws were regularly dissolved at a corrosion rate of 0.014 mm/year without the production of any gas bubbles, which make this alloy superior to magnesium-based biodegradable materials. However, the degradation should be enhanced to fulfil the basic criteria for absorbable implants. The material did not induce any inflammatory reaction locally (around the experimental screw) or distally, e.g., in the vital organs such as the liver, kidneys, and brain. Neither bone resorption nor systemic toxicity were observed. Periosteal apposition and endosteal remodelling were observed in the implant vicinity. More than 50% of implant surfaces were covered by fibrous tissue, while osteolysis of the surrounding bone was not observed. Histologically, it was proven that the experimental alloy represents a promising prospect for future applications of Zn-based materials in clinical use.

## Figures and Tables

**Table 1:** A summary of the results of the in-vivo tests on Zn/Mg alloys performed by various scientific groups.

No. of Animals, Experimental Time	Setting	Zn/Mg Alloy	Methods of Analysis	Results	Ref.
30 rats, 4, 8, 12 weeks	Femoral condyle defect, with 99.99% Ti (N = 15), Zn–0.8Sr (wt.%), N = 15	Zn–0.8Sr (wt.%) Ti 99.99%	microCT, Histomorphometry, SEM	Good biosafety. Osteogenesis time related: greater after 12 weeks than after 4 weeks. Biodegradation products greater around Zn–0.8Sr alloy. Heart, liver, spleen, lung, and kidneys—no abnormalities in the Zn–0.8Sr alloy group in comparison with the pure Ti group. The concentration of Zn^2+^ and Sr^2+^ in the blood and organs of the Zn–0.8Sr group was not higher than the pure Ti implant group	[22]
54 rats, 8 weeks	Experimental alloys implanted into femoral bone of rats	Zn 99.99% Zn–0.1Sr Zn–0.1Mn Zn–0.4Cu Zn–0.4Fe Zn–0.2Li Zn–0.8Mg Zn–0.8Ca Zn–2.0Ag	microCT, Histology, SEM, blood analysis of Zn concentration	No gas, no obvious degradation after 8 weeks. Circumferential osteogenesis. Volume of pure Zn decreased to 95.12 ± 1.39% after 8 weeks and degradation rate was 0.14 ± 0.05 mm/year. Zn–0.4Cu alloys had higher rate of degradation: 0.26 ± 0.03 mm/year. Faster degradation: Zn–2.0Ag, Zn–0.4Li, Zn–0.4Fe, and Zn–0.8Mg. Same degradation rate as pure Zn: Zn–0.1Mn, Zn–0.8Ca, and Zn–0.8Sr	[23]
27 beagle dogs, 8, 12, 24 weeks	Experimental alloy used for treatment of mandibular fractures of beagle dogs. Control group: Ti 99.99%, PLLA (poly-l-lactic acid)	Zn–2.0Mg–2.0Fe PPLA Ti 99.99%	3-point bending test, X-ray, microCT, histology, analysis of Zn concentration in blood, before surgery, 4, 12, and 24 weeks after surgery	Zinc alloy: good mechanical properties. Increasing in vivo degradation rate of the zinc alloy implants: 0.033 ± 0.015 mm/year at 4 weeks, 0.079 ± 0.009 mm/year at 12 weeks, 0.095 ± 0.009 mm/year at 24 weeks. Good biocompatibility: no difference between the pre-op and post-op blood and biochemical results. Serum zinc value in the zinc alloy group was slightly higher after implantation than before, no statistically significant difference	[24]
12 rabbits, 24 operated sites, 16 weeks	Fracture of ulnar bone and its osteosynthesis	99.9% Mg (wt.%)	Mechanical testing, X-ray, microCT, histology	Well tolerated. Corrosion product formation and gas formation. Histologically normal bone properties. All fractures healed. Bone growth over and around degrading Mg devices. Good mechanical properties of healed bone. Facilitated fracture healing while stimulating local bone growth	[3]
32 rats, 24 weeks	Femoral pin implanted bilaterally	ZX50 *, WZ21 **	Histology, microCT	Volume loss: ZX50: 1.2%/day, WZ21: 0.5%/day. ZX50: massive gas production. WZ21: new bone production, good osteoconductivity and osteoinductivity of Mg. Excellent bone recovery	[2]
36 rats, 4, 8, 12 weeks	Ulnar bone defect replaced by a metallic experimental alloy tube	Mg–3Zn–1Ca–0.5REE ***/ hydrothermally (HT) coated and uncoated alloy	Histology, microCT, X-ray, serum Mg^2+^ and Ca^2+^ concentrations	Mg^2+^ normal levels in all groups. Ca^2+^ levels higher than normal, but not significantly. No organ histological pathology. HT coated alloy with better biocompatibility and higher resistance to corrosion	[11]
8 rabbits, 9 months to 3.5 years	Cylindrical alloy implant in a medullary cavity	LAE442 ****	Histology, microCT, mechanical testing, organ corrosion products testing	Good biocompatibility, no gas formation, no toxicity in vital organs. Slow resorption: 2–2.5% in 9 months, 5% in 3.5 years. Accumulation of REE *** in implant site and vital organs	[12]
18 rabbits, 16 weeks	Alloy plate and 2 screws implanted into femoral bone	99.9% Mg Mg–1Sr (wt.%)	Histology, blood count, Sr concentration in implanted site and vital organs	Corrosion rate slower in group with Sr. Good biocompatibility in the group with Sr. Highest increase of Sr^2+^ concentration in liver (388 ± 25 μg/kg) at 4w post-implantation and returned to 32 ± 2 μg/kg after 16 weeks implantation. Increase in the Sr^2+^ ion concentration in the blood from 2–8 weeks post-implantation	[25]
48 humans, 12 months	Treatment of osteonecrosis of femoral head by vascularized bone grafting. Two groups: (i) Mg screw (ii) without fixation	99.9 Mg (wt.%) ^§^	X-ray, CT, functional recovery, Harris hip score (HHS), serum levels of Mg, Ca, and P	Mg screw group 25% volume reduction in 12 months. Normal levels of Mg, Ca, P in both groups. HHS was significantly improved in the Mg screw group	[26]
23 goats, 4, 8, 12, 48 weeks	Femoral neck fractures. Control group: no treatment/empty defect	Mg 99.99 wt.% ^§^	Histology, microCT, X-ray, serum Mg and Ca ion, liver and kidney functions pre-op and at 2, 4, 8, 12, and 48 weeks after surgery.	Normal levels of ions all weeks/all groups. Normal organ function & histology. All fractures healed. Degradation of metal: 10% at 4 weeks, 38.8% at 12 weeks and 45.3% at 48 weeks. No cytotoxicity. Good bone production around screws. No gas production observed	[27]
36 rabbits, 6, 12, 24 weeks	Reconstruction of anterior cruciate knee ligament by Mg alloy and titanium screws	MgYREEZr and Ti–6Al–4V (wt.%)	Histology, microCT, blood testing for alloy elements	Good biocompatibility, no inflammatory changes, no necrosis. Similar results to titanium alloy. Gas production decreased within 24 weeks. Very low levels of alloy elements in blood	[28]
18 rats, 4, 26, and 52 weeks	Pins into femoral bone of a rat, one group treated by ZX10, other by ZX20	ZX10 and ZX20 ^§§^ ZX20	Histology, microCT	Higher degradation rate of ZX20. Histologically ZX10 and ZX20 are well tolerated. Good implant-bone contact from 4 weeks post-op Gas production: insignificant between pure Mg (99.999%) and ZX10, significantly higher gas production of ZX20, compared to ZX10 and pure Mg	[29]

* ZX50 = Mg–5.0Zn–0.25Ca–0.15Mn (wt.%)—early degradation. ** WZ21 = Mg–1.0ZN–0.25Ca–0.15Mn (wt.%)—longer degradation. *** Rare earth elements (REE). **** LAE442: LAE442 (Mg–4%Li–3.6%Al–2.4%REE. ****, in wt.%) ^§^ Mg 99.99%; Al 0.002; Si < 0.001; Ca < 0.001; Ti < 0.0001; Mn 0.002; Fe 0.001; Ni < 0.0001; Cu 0.0002; Zn 0.0028; Pb 0.0008 (wt.%). MgYREEZr: Mg–4.3Y–0.4Zr–4.4REE (wt.%). ^§§^ ZX10: Mg‒1.0Zn‒0.3Ca (in wt.%)/ZX20: Mg‒1.5Zn‒0.25Ca (in wt.%).

**Table 2:** Evaluation of the healing process according to parameters on histology of specimens (Reifenrath et al.) [35].

Parameter	Score	Interpretation	120 Days of Implantation						
Gas bubbles	0	No	0	0	0	0	0	0	NI
—	1	Yes	—	—	—	—	—	—	—
Overall impression of bone structure (BS)	0	Smooth	0	0	—	—	0	—	—
	1	Irregular	—	—	1	1	—	1	NI
Bone cavities (BC)	0	≤3 osteon-like cavities	0	0	0	0	0	0	NI
	1	4–6 osteon-like cavities or ≤10 smaller	—	—	—	—	—	—	—
	2	7–10 osteon-like cavities or 11–20 smaller	—	—	—	—	—	—	—
Periosteal remodelling (PR)	0	No	—	—	NA	1	—	—	NI
	1	≥1/4 periosteal bone 1 osteon thick	2	2	—	—	2	2	—
	2	≥1/4 periosteal bone 2 osteon thick	—	—	—	—	—	—	—
	3	≥1/4 periosteal bone 3 osteon thick	—	—	—	—	—	—	—
Endosteal remodelling (ER)	0	No	—	0	—	NC	NC	NC	NI
	1	≥1/4 endosteal bone 1 osteon thick	—	—	—	—	—	—	—
	2	≥1/4 endosteal bone 2 osteons thick	—	—	2	—	—	—	—
	3	≥1/4 endosteal bone 3 osteons thick	3	—	—	—	—	—	—
Periosteal apposition (PA)	0	No	—	—	NA	—	—	—	NI
	1	Yes	1	1		1	1	1	—
Peri-implant bone formation (PIF)	0	No	—	—	—	—	—	—	NI
	1	Yes	1	1	NI	1	1	1	
Peri-implant fibrosis (PF)	0	No	—	—	—	—	—	—	NI
	1	≤25% implant surface	—	—	—	—	—	—	—
	2	25–50% implant surface	—	—	—	—	2	—	—
	3	≥51% implant surface	3	3	NI	3	—	3	—
	[mm]	max. thickness	0.10	0.10	0.05	0.04	0.05	0.05	
Lymphoplasmacellular reaction (LYM)	0	<30 cells per section	0	0	0	0	0	0	NI
	1	30–50 cells per section	—	—	—	—	—	—	—
	2	51–100 cells per section	—	—	—	—	—	—	—
	3	>100 cells per section	—	—	—	—	—	—	—
Macrophages (MPH)	0	<3 cells per section	—	0	0	0	0	0	NI
	1	3–20 cells per section	1	—	—	—	—	—	—
	2	>20 cells per section	—	—	—	—	—	—	—
Giant cells (GC)	0	No	0	0	0	0	0	0	NI
	1	1–10 cells per section	—	—	—	—	—	—	—
	2	>10 cells per section	—	—	—	—	—	—	—
Interface—features of material corrosion	0	No	0	0	0	0	0	0	0
	1	Yes	—	—	—	—	—	—	—

Note: NA = not applicable, NI = no implant visible, NC = not applicable a screw in compacta.

**Table 3:** Organ analysis of Magnesium, Zinc and Strontium content, mean ± SMODCH.

	Experimental Group—This Study			Control Groups (For Details See References)		
Tissue	Zinc [mg/kg] of Tissue	Magnesium [mg/kg] of Tissue	Strontium [μg/kg] of Tissue	Zinc [mg/kg] of Tissue	Magnesium [mg/kg] of Tissue	Strontium [μg/kg] of Tissue
Brain	10.4 ± 0.99	144 ± 5	120 ± 35	~11.3 [40] ~11.0 [41] ~16.7 [42]	~152 [40] ~257 [42]	-
Kidneys	24.27 ± 1.18	177 ± 14	50 ± 5	~23.4 [40] ~10.2 [43] ~44.3 [42]	~182 [40] ~345 [42]	~19 [25]
Liver	31.57 ± 1.97	174 ± 6	28 ± 19	~32.1 [40] ~24.6 [41] ~14.7 [43] ~41.9 [42]	~151 [40] ~349 [42]	~32 [25]

## Data Availability

The data presented in this study are available on request from the corresponding author.
